# 14-3-3 Protein of *Neospora caninum* Modulates Host Cell Innate Immunity Through the Activation of MAPK and NF-κB Pathways

**DOI:** 10.3389/fmicb.2019.00037

**Published:** 2019-01-25

**Authors:** Shan Li, Pengtao Gong, Nan Zhang, Xin Li, Lixin Tai, Xu Wang, Zhengtao Yang, Ju Yang, Xingquan Zhu, Xichen Zhang, Jianhua Li

**Affiliations:** ^1^Key Laboratory of Zoonosis Research by Ministry of Education, Institute of Zoonosis, College of Veterinary Medicine, Jilin University, Changchun, China; ^2^State Key Laboratory of Veterinary Etiological Biology, Key Laboratory of Veterinary Parasitology of Gansu Province, Lanzhou Veterinary Research Institute, Chinese Academy of Agricultural Sciences, Lanzhou, China

**Keywords:** *Neospora caninum*, 14-3-3, immune protection, MAPK, AKT, cytokines

## Abstract

*Neospora caninum* is an obligate intracellular apicomplexan parasite, the etiologic agent of neosporosis, and a major cause of reproductive loss in cattle. There is still a lack of effective prevention and treatment measures. The 14-3-3 protein is a widely expressed acidic protein that spontaneously forms dimers within apicomplexan parasites. This protein has been isolated and sequenced in many parasites; however, there are few reports about the *N. caninum* 14-3-3 protein. Here, we successfully expressed and purified a recombinant fusion protein of Nc14-3-3 (rNc14-3-3) and prepared a polyclonal antibody. Immunofluorescence and immunogold electron microscopy studies of tachyzoites or *N. caninum*-infected cells suggested that 14-3-3 was localized in the cytosol and the membrane. Western blotting analysis indicated that rNc14-3-3 could be recognized by *N. caninum*-infected mouse sera, suggesting that 14-3-3 may be an infection-associated antigen that is involved in the host immune response. We demonstrated that rNc14-3-3 induced cytokine expression by activating the MAPK and AKT signaling pathways, and inhibitors of p38, ERK, JNK, and AKT could significantly decrease the production of IL-6, IL-12p40, and TNF-α. In addition, phosphorylated nuclear factor-κB (NF-κB/p65) was observed in wild-type peritoneal macrophages (PMs) treated with rNc14-3-3, and the protein level of NF-κB/p65 was reduced in the cytoplasm but increased correspondingly in the nucleus after 2 h of treatment. These results were also observed in deficient in TLR2^-/-^ PMs. Taken together, our results indicated that the *N. caninum* 14-3-3 protein can induce effective immune responses and stimulate cytokine expression by activating the MAPK, AKT, and NF-κB signaling pathways but did not dependent TLR2, suggesting that Nc14-3-3 is a novel vaccine candidate against neosporosis.

## Introduction

*Neospora caninum* is an obligate intracellular parasite and one of the most important infectious causes of abortion in cattle worldwide; this pathogen is closely related to *Toxoplasma gondii* (Almeria et al., 2016). There is no evidence of *N. caninum* infectivity in humans, but serological evidence suggests that humans can be exposed to *N. caninum* (Tranas et al., 1999). Neosporosisis less clinically and economically important than toxoplasmosis; however, increasing evidence suggests that *N. caninum* is an important cause of abortions in small ruminants and may even be the main cause of reproductive losses in cows (Dubey, 2003). Thus, neosporosis leads to significant economic losses in both the dairy and beef industries worldwide.

Many control measures have been proposed to reduce *N. caninum* infection, including drug treatment and vaccination; however, there is still a lack of effective and safe prevention or treatment strategies. To replicate, *N. caninum* actively invades host cells, including immune cells, and leads to altered interactions with host immune mechanisms (Reid et al., 2012). The host induces the immune response and the production of inflammatory cytokines to eliminate infections by the recognition of highly conserved sets of pathogen-associated molecular patterns (PAMPs) through pattern-recognition receptors (PRRs). Previous studies have shown that the Toll-like receptor 2 (TLR2) innate recognition pathway triggers an inflammatory response to control *N. caninum* infection (Mota et al., 2016). However, which PAMPs or antigens participate in the activation of innate immunity by *N. caninum* is not yet known. Therefore, a better understanding of the immune mechanisms that mediate host resistance to neosporosis may facilitate the discovery of approaches to control neosporosis.

The 14-3-3 proteins are a family of highly conserved scaffolding proteins that are widely expressed in all eukaryotic cells and have been implicated in the regulation of a variety of important cellular processes, such as the cell cycle, apoptosis, and mitogenic signaling (Tian et al., 2015). In *Eimeria tenella*, 14-3-3 proteins are involved in the regulation of the mannitol cycle metabolic pathway, which is believed to be an essential energy source for the sporulation process, and 14-3-3 proteins are a potential target for interrupting the *E. tenella* life cycle (Liberator et al., 1998). In *Schistosoma japonicum* and *Schistosoma mansoni*, 14-3-3 proteins are known to regulate PKC activity and translocation during the parasite life cycle (Zhang et al., 2000; Siles-Lucas Mdel and Gottstein, 2003). The *T. gondii* 14-3-3 protein has been proved to be a novel vaccine candidate against toxoplasmosis (Meng et al., 2012). However, the characterization and immunomodulatory effects of *N. caninum* 14-3-3 proteins have not yet been reported.

Here, we present the first characterization and immunomodulatory investigation of the *N. caninum* 14-3-3 protein. We successfully expressed and purified a recombinant fusion protein of Nc14-3-3 (rNc14-3-3), and showed that rNc14-3-3 could induce the activation of the AKT, MAPK, and nuclear factor-κB (NF-κB)/p65 signaling pathways in mouse peritoneal macrophages (PMs) independent of TLR2. In addition, our study indicated that the rNc14-3-3 protein can induce effective immune responses and stimulate cytokine expression through the MAPK and AKT signaling pathways, suggesting that rNc14-3-3 is a novel vaccine candidate against neosporosis.

## Materials and Methods

### Animals

Female C57BL/6 mice (6–8 weeks old) were purchased from the Changsheng Experimental Animal Center (Changchun, China), and TLR2-deficient (TLR2^-/-^) mice were obtained from the Model Animal Research Center of Nanjing University (Nanjing, China). The mice were supplied with sterile food and water and maintained under specific pathogen-free conditions in the National Experimental Teaching Demonstration Center of Jilin University (Changchun, China).

### Parasites and Cell Culture

*Neospora caninum* tachyzoites (Nc-1 strain) were maintained by serial passages in VERO cells in Roswell Park Memorial Institute (RPMI) 1640 medium supplemented with 2% fetal bovine serum (FBS) (Biological Industries, Ltd., Beit HaEmek, Israel), 2 mM L-glutamine, 100 U of penicillin, and 100 μg of streptomycin (P/S) (all from Life Technologies, Carlsbad, CA, United States) in a 5% CO_2_ atmosphere at 37°C. Free *N. caninum* tachyzoites were obtained and harvested from VERO cells [centrifuged at 500 ×*g*/min for 10 min at room temperature (RT)] and then passed through a 1-ml syringe and centrifuged at 1,500 ×*g* for 30 min to remove host cell debris by gradient density centrifugation with a 40% Percoll (GE Healthcare, Uppsala, Sweden) solution (v/v). The parasite pellet was collected and washed twice with RPMI 1640, and the *N. caninum* concentration was determined in a hemocytometer.

The wild-type (WT) and TLR2^-/-^ mice were inoculated intraperitoneally with thioglycollate medium (BD Biosciences, New Zealand, United States) for 4 days, and PMs were harvested in cold phosphate-buffered saline (PBS) as previously described (Li et al., 2017; Wang et al., 2017). In addition, PMs were cultured in complete RPMI 1640 medium supplemented with 10% FBS, 100 U/ml penicillin, 100 U/ml streptomycin and 2 M L-glutamine.

### Cloning, Expression, and Purification of rNc14-3-3

The total DNA of *N. caninum* was extracted from purified tachyzoites according to the manufacturer’s protocol. The 14-3-3 gene [GenBank: U31542.1] was amplified by polymerase chain reaction (PCR) using the primers 14-3-3-F (5′-CGC*GGATCC*ATGGCGGAGGAAATCAAAAATCT-3′) and 14-3-3-R (5′-CCG*GAATTC*CTGATCAGCCTGCTCAGCGG-3′), which contained *Bam HI* and *EcoR I* sites (underlined), respectively. The obtained 801-bp PCR product that corresponded to 14-3-3 was digested with *Bam HI*/*EcoR I* and cloned into the corresponding sites of the pGEX-4T-1 vector. The positive recombinant plasmid pGEX-Nc14-3-3 was transformed into the *E. coli* expression strain Rosetta DE3a (TIANGEN, Beijing, China), and glutathione *S*-transferase (GST) fusion protein expression was induced with 0.1 mM isopropyl-β-D-1-thiogalactopyranoside (IPTG) for 4 h at 37°C. The protein was then solubilized and purified with Proteinlso GST Resin (TransGen Biotech, Beijing, China) according to the manufacturer’s protocol. Briefly, after sonication and centrifugation, the supernatant was filtered through a 0.45-μm membrane and loaded slowly into a 2.5 cm × 10 cm column in which the GST-Bind resin had been equilibrated with 10 column volumes of binding buffer (140 mM NaCl, 2.7 mM KCl, 10 mM Na_2_HPO_4_, and 1.8 mM KH_2_PO_4_, pH 7.3). The column was then washed with the wash buffer. Finally, the fusion protein GST-14-3-3 was eluted with elution buffer (50 mM Tris-HCl, pH 8.0, and 10 mM reduced-glutathione). The purified protein was dialyzed in PBS for at least 5 h at 4°C with two buffer changes. To remove possible contaminants from the process, purification was performed as previously described, with a slight modification (Liu et al., 1997; Macedo et al., 2013). Briefly, Triton X-114 was added to the purified protein fraction to a final concentration of 1%. The mixture was incubated at 4°C for 10 min, with constant stirring to ensure a homogenous solution, and then transferred to a 37°C water bath and incubated for 10 min until oily matter appeared; the sample was then centrifuged at 13,000 ×*g* for 10 min (RT). The upper aqueous phase was removed and subjected to additional Triton X-114 phase separation two times. Finally, the upper water phase containing the protein was collected carefully and stored at -80°C until use. In addition, *N. caninum* lysate antigen (NLA), excretory secretory antigens (ESAs) and extracellular vesicles (EVs) were prepared as previously described (Li et al., 2018). The collected proteins were either stored at -80°C or directly used in additional experiments. The pellets were resuspended in protein loading buffer and stored at -20°C or immediately analyzed. All protein concentrations were measured using the BCA Protein Assay Kit (Thermo Scientific, Waltham, MA, United States).

To assess the level of endotoxin in rNc14-3-3 proteins, the ToxinSensor^TM^ chromogenic LAL Endotoxin Assay Kit (GenScript, Piscataway, NJ, United States) was used according to the manufacturer’s instructions.

### Production of Antibodies

Mouse polyclonal antibodies against Nc14-3-3 were prepared according to previously described protocols (Dong et al., 2017). Briefly, six 8-week-old BALB/C mice were subcutaneously immunized with rNc14-3-3 protein (100 μg/mouse), which was emulsified with Freund’s complete adjuvant (Sigma, St. Louis, MO, United States). Boosters were administered at 2, 4, and 6 weeks with rNc14-3-3 protein (50 μg/booster), which was emulsified with Freund’s incomplete adjuvant (Sigma, St. Louis, MO, United States). One week after the last injection, mouse blood was collected via orbital bleeding, and the sera were obtained. The levels of antibodies in mouse sera were detected by enzyme-linked immunosorbent assay (ELISA) as follows. Briefly, 96-well microtiter plates were coated with 1 μg of SNAg in 100 μl of carbonate buffer (150 mM Na_2_CO_3_, 349 mM NaHCO_3_, pH 9.6) and incubated at 4°C overnight. After washing three times, the plates were blocked with 3% bovine serum albumin (BSA) for 2 h at 37°C and subsequently incubated with mouse sera diluted in PBS for 2 h at 37°C. HRP-conjugated goat anti-mouse IgG antibody (Proteintech, Wuhan, China) was used as a secondary antibody at a 1:2000 dilution, and the reaction were revealed by TMB (Beyotime, Shanghai, China) for 15 min at 37°C and stopped by 2 M H_2_SO_4_. The reaction was measured at 490 nm with an ELISA reader. Finally, the generated mouse polyclonal antibodies were purified using HisTrap for protein G (GE Healthcare Bio-Sciences, Ltd., Marlborough, MA, United States) according to the instructions.

### Immunofluorescence

Indirect immunofluorescence assays (IFAs) were performed in both extracellular and intracellular *N. caninum* tachyzoites. Free *N. caninum* tachyzoites (Nc-1) were washed three times with PBS, added to poly-L-lysine (0.1 mg/ml, Sigma Aldrich) precoated cover glass slides, dried at RT and fixed in 4% paraformaldehyde for 10 min. The cover slides were then washed three times with PBS, permeabilized with 0.25% Triton X-100 in PBS for 10 min, washed and blocked in 3% BSA/PBS for 2 h at RT. After blocking, the samples were incubated with a 1:100 dilution of the 14-3-3 antibody overnight at 4°C, then washed and incubated with the secondary fluorescein isothiocyanate (FITC)-conjugated goat anti-mouse Ig-G (Proteintech, Wuhan, China) for 1 h at RT. The coverslips were stained with 4′,6-diamidino-2-phenylindole (DAPI) (Thermo Scientific, Waltham, MA, United States) for 10 min before analysis on an Olympus FV1000 Laser Scanning Confocal microscope (Japan).

*Neospora caninum* tachyzoite-infected VERO cells were stained with the 14-3-3 antibody as follows. The intracellular tachyzoites were washed with cold PBS three times and fixed on glass coverslips with 4% formaldehyde for 20 min at 4°C. The infected cells were permeabilized with 0.25% Triton X-100 in PBS for 8 min, washed and blocked in 3% BSA/PBS for 2 h at RT. All procedures were the same as those described above.

### Immunogold Electron Microscopy

Free *N. caninum* tachyzoites or VERO cells challenged with *N. caninum* tachyzoites for 12 h (MOI = 1:1, parasite:cell) were collected and fixed with 4% paraformaldehyde (pH 7.2) for 2 h. Then, the samples were dehydrated with 50, 70, 90, and 100% *N,N*-Dimethylformamide (DMF) for 15 min at 4°C in sequence, incubated with mixtures DMF: LR White Resin = 2:1 and DMF: LR White Resin = 1:2 for 30 min at 4°C in sequence. Then, they were incubated with LR White Resin overnight at 4°C, replaced fresh LR White Resin, embed and UV irradiation polymerization for 10 days at -20°C. The samples were washed, cut into 80-nm sections and applied to a carbon-coated copper grids and stored at 4°C. For immunolabeling, the grids were blocked in 3% BSA/PBS for 30 min at RT; then, the 14-3-3 antibody (1:100) was diluted in 1% BSA/PBS and added to the grid for 40 min. Following washes with PBS, the secondary antibody conjugated to 10-nm colloidal gold (Jackson ImmunoResearch, West Grove, PA, United States) was used at a 1:100 dilution and incubated for 40 min. The grids were examined used a Hitachi 7650 transmission electron microscope operating at an accelerating voltage of 80 kV. Images were obtained using an Olympus SIS Megaview II digital camera and iTEM software.

### Mouse PMs Viability Assay

We examined the effect of rNc14-3-3 on PMs viability using Cell Counting Kit (CCK-8) (Dojindo Laboratories, Japan) according to manufacturer’s directions. The mouse PMs were plated in 96-well plates at the density of 1 × 10^4^ and incubated for 24 h. Then cells were stimulated with rNc14-3-3 at concentration of 12.5, 25, 50, 100, 200 ug/ml in 100 μl sterile PBS or PBS alone, respectively. After incubation for 24 h, 10 μl of CCK-8 reagent was added to each well and incubation continued for another 3 h. Finally, the absorbance was measured at 450 nm.

### Cellular Fractionation

The cytoplasmic and nuclear proteins were extracted using a Nuclear and Cytoplasmic Protein Extraction Kit (KeyGen Biotech, Nanjing, China) according to the instructions. Briefly, 6 × 10^6^ cells were collected in 1.5 ml tubes and washed twice with ice-cold PBS and resuspended in 450 μl of prechilled Buffer A with 50 μl of Buffer B, then incubated on ice for 30 min. The cell lysates were centrifuged at 800 ×*g* for 10 min, and the resulting supernatants were the cytoplasmic fraction. The pellets were lysed in 100 μl of Buffer C (17 μl of 100 mM PMSF and 1 μl of protease inhibitor/1 ml of Buffer C), vortexed strongly for 15 s and then incubated on ice for 60 min. The lysates were then centrifuged at 13,000 ×*g* for 30 min, and the resulting supernatants were the nuclear fraction.

### Western Blotting Analysis

The PMs were seeded in 6-well culture plates (Corning Incorporated, Corning, NY, United States) at a density of 3 × 10^6^ cells/well and treated with rNc14-3-3 for the indicated times. Then, the cell lysates were extracted from PMs using a total protein extraction kit (KeyGen Biotech, Nanjing, China). Protein concentrations were measured using the BCA Protein Assay Kit (Thermo Scientific, Waltham, MA, United States). Thirty micrograms of each protein sample was separated via 12% sodium dodecyl sulfate-polyacrylamide gel electrophoresis (SDS-PAGE) and then transferred to polyvinylidene fluoride (PVDF) membranes (Millipore, Bedford, MA, United States). After 2 h of blocking in TBS-0.05% Tween 20 containing 5% skim milk, the membranes were incubated at 4°C overnight with the following antibodies: mouse anti-14-3-3 and primary rabbit antibodies against p-P38 (1:1,000), total P38 (1:1,000), p-ERK1/2 (1:1,000), total ERK1/2 (1:1,000), p-JNK (1:1,000), total JNK (1:1,000), p-AKT (1:1,000), total AKT (1:1,000), p-IκB (1:1,000), total IκB (1:1,000) (all from Cell Signaling Technology, United States); p-p65 (1:1,000), total p65 (1:1,000) (from Abcam, United Kingdom); and GAPDH (1:1,000). For all secondary antibody incubations, horseradish peroxidase (HRP)-conjugated goat anti-mouse or goat anti-rabbit antibodies (Proteintech, Wuhan, China) were used at a 1:2000 dilution. The membranes were visualized by an enhanced chemiluminescence (ECL) Western Blot Detection System (Clinx Science Instruments, Co., Ltd., Shanghai, China).

### AKT Knockdown

To further confirm the role of the AKT signaling pathway in mouse PMs after treatment with rNc14-3-3, a small-interfering RNA (siRNA) specific for AKT was designed and synthesized by Ruibo Biology Company (Guangzhou, China). The mouse PMs were seeded in 6-well culture plates at a density of 3 × 10^6^ cells/well and transfected with siRNA-control and siRNA-AKT for 24 h using Lipofectamine 2000 (Invitrogen, Carlsbad, CA, United States) following the manufacturer’s protocol. To detect interference effects, the cells were collected for mRNA and protein analyses. In the formal experiment, after transfection, the cells were further stimulated with 50 μg/ml rNc14-3-3 for 24 h. The supernatants were harvested for cytokine measurement.

### Enzyme-Linked Immunosorbent Assay

The PMs were seeded in 24-well culture plates (Corning Incorporated, Corning, NY, United States) at a density of 5 × 10^5^ cells/well and treated with rNc14-3-3. In some experiments, the PMs were pretreatment for 2 h with a p38 inhibitor (SB203580; 30 μM) (Sigma, St. Louis, MO, United States), for 1 h with an ERK inhibitor (PD98059; 40 μM) (Sigma), for 1 h with a JNK inhibitor (SP600125; 10 μM) (Sigma), or for 1 h with an AKT inhibitor (IV; 10 μM) (Santa Cruz Biotechnology, United States); each well was incubated in the presence or absence of a p38, ERK, or JNK inhibitor or PBS before rNc14-3-3 treatment. The concentration of each inhibitor was chosen based on previously published manuscripts (Jin et al., 2017b; Li et al., 2017). After 24 h of treatment with rNc14-3-3, the supernatants were harvested for cytokine measurement, and Cytokine ELISA Ready-SET-Go kits (eBioscience, San Diego, CA, United States) were used to detect IL-12p40, TNF-α, and IL-6 levels following the manufacturer’s instructions. The assays were read at 450 nm, and the optical density (OD) values obtained were converted to pg/ml by extrapolation using a standard curve.

### Subcellular Localization of NF-κB

To determine the localization of NF-κB/p65, 5 × 10^5^ PMs were seeded in 24-well culture plates (Corning Incorporated, Corning, NY, United States) with sterile glass slides, and the cells were treated with rNc14-3-3 (50 μg/ml) or Pam3CSK4 (TLR2/TLR1 Agonist, InvivoGen, United States, 10 ug/ml) at 37°C. After 30 min, the cells were washed twice with sterile PBS, fixed with 4% paraformaldehyde for 15 min, permeabilized with 0.25% Triton X-100 in PBS for 10 min, and washed. Then, the cells were incubated with a rabbit anti-phospho-NF-κB/p65 antibody (Santa Cruz, CA, United States) at a 1:100 dilution at 4°C overnight. The cells were washed and incubated with FITC-conjugated goat anti-rabbit IgG (Proteintech, Wuhan, China) for 1 h at RT. The cells were washed, and the coverslips were stained with DAPI before analysis on a Zeiss LSM 710 confocal microscope equipped with a 633, 1.4-NA, oil-immersion objective (Carl Zeiss).

### Statistical Analysis

Data were expressed as the standard error of the mean ± standard deviation (SD), and the means were compared by one-way analysis of variance using SPSS 19.0 software (SPSS, Inc., Chicago, IL, United States). All graphs were generated in GraphPad Prism 7.00, and independent experiments were performed with at least three technical replicates.

## Results

### Characteristics and Localization of the Nc14-3-3 Protein

**FIGURE 1:**
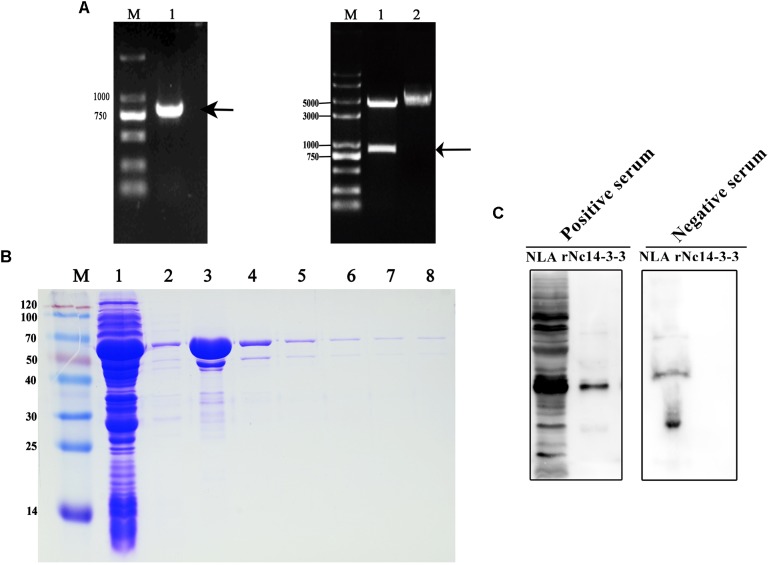
Constructed plasmids and purified recombinant Nc14-3-3 protein. **(A,** Left) The Nc14-3-3 gene were amplified from cDNA using PCR. lane M: DNA Mark, lane 1: an 801-bp PCR product, arrow: an 801-bp PCR product that corresponded to Nc14-3-3. (Right) The product was cloned into the vector pGEX4T-1 and identified through restriction enzyme analysis. lane M: DNA Mark, lane 1: the pGEX-4T-1-Nc14-3-3 vector was confirmed by digestion with *EcoRI* and *BamHI*, lane 2: the pGEX-4T-1-Nc14-3-3 vector without digestion. **(B)** rNc14-3-3 purified using the Proteinlso GST Resin. M, protein molecular markers. Lanes 1–2, SDS-PAGE analysis of effluent and washed liquid. Lanes 3–8, the eluted buffer. **(C)** Western blotting analyzed of *Neospora caninum* lysate antigen (NLA) and rNc14-3-3 using anti-*N. caninum* serum (1:200). positive serum from mouse infected with *N. caninum*; negative serum from healthy mouse.

An 801-bp PCR product was generated that corresponded to Nc14-3-3 (Figure 1A, left); the product was digested with *Bam HI*/*EcoR I* and cloned into the corresponding sites of the pGEX-4T-1 vector. To confirm the process, we detected the product by PCR and restriction enzyme analysis. The results of the restriction enzyme digestion of plasmids are shown that we have successfully constructed expression plasmid (Figure 1A, right). To further assess the localization of Nc14-3-3, a recombinant fusion protein of Nc14-3-3 (rNc14-3-3) was successfully expressed and purified using a GST Resin (Figure 1B). Western blotting analysis showed good immunoreactivity of rNc14-3-3 to serum (1:200) from a mouse infected with *N. caninum* (Figure 1C).

**FIGURE 2:**
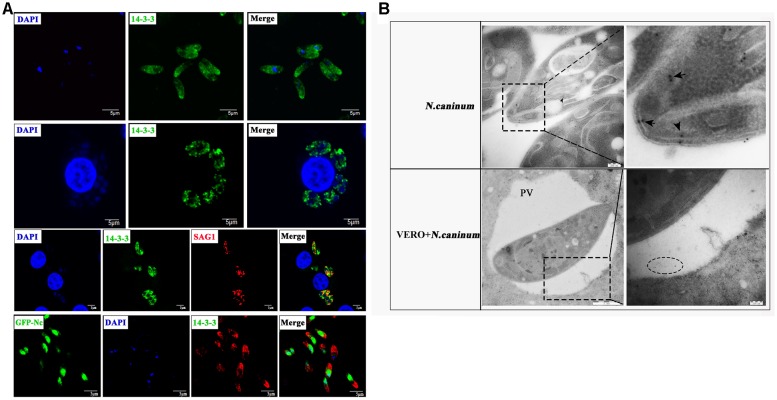
Immunofluorescence **(A)** and immunogold **(B)** studies of 14-3-3 protein localization. **(A)** Immunofluorescence microscopy of *N. caninum* tachyzoites or VERO cells infected with *N. caninum*. Scale bars: 5 μm. **(B)** Immunogold labeling of VERO cells infected with *N. caninum* tachyzoites. arrow: the gold particles within the tachyzoites, circle: the gold particles within the perivacuolar space. PV, parasitophorous vacuole.

Next, we detected the localization of 14-3-3 in *N. caninum* tachyzoites. Free *N. caninum* tachyzoites and VERO cells infected with *N. caninum* tachyzoites were subjected to immunofluorescence analysis using the polyclonal anti-*N. caninum* 14-3-3 antibody, and the results showed that the 14-3-3 protein was mainly found in the cytosol and cell membrane (Figure 2A). To obtain additional information, we used electron microscopy, which allows ultrastructural observations of the cells, and immunogold labeling of 14-3-3 protein. 14-3-3 was detected in the cytosol and cell membrane, in agreement with the immunofluorescence results. In addition, VERO cells infected with *N. caninum* tachyzoites showed that 14-3-3 was also seemingly to present in the parasitophorous vacuole (PV) (Figure 2B), certainly, this conclusion still required further evidence.

### Effect of rNc14-3-3 on the Cell Viability of Mouse PMs *in vitro*

**FIGURE 3:**
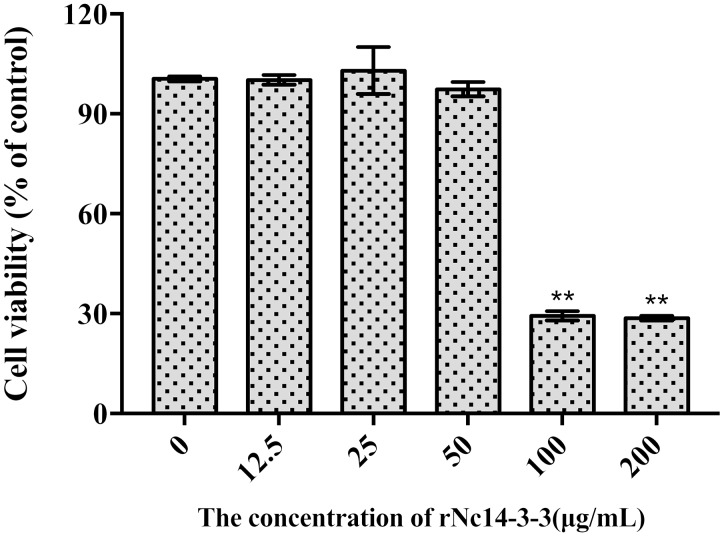
Effect of Recombinant Nc14-3-3 on the cell viability of PMs. Mouse PMs were cultured with different protein concentrations (0, 12.5, 25, 50, 100, 200 μg/mL) for 24 h. Cell viability was determined by the CCK-8 assay. ^∗^*P* < 0.05; ^∗∗^*P* < 0.01 for the rNc14-3-3 group versus the PBS group. CCK-8, cell counting kit.

To determine the appropriate concentration of rNc14-3-3 used in our experiments, we investigated the influence of it on the viability of Mouse PMs using CCK-8 assay. As shown in Figure 3, rNc14-3-3 did not affect the cell viability at concentrations lower than 50 μg/mL. However, the cell viability was significantly reduced at the concentration of 100 μg/mL (*P* < 0.05 vs. untreated cells). Thus, the appropriate concentration of rNc14-3-3 *in vitro* for Mouse PMs was 50 μg/ml.

### Recombinant Nc14-3-3 Activates MAPK Signaling Pathways in Mouse PMs in a Manner Independent of TLR2

**FIGURE 4:**
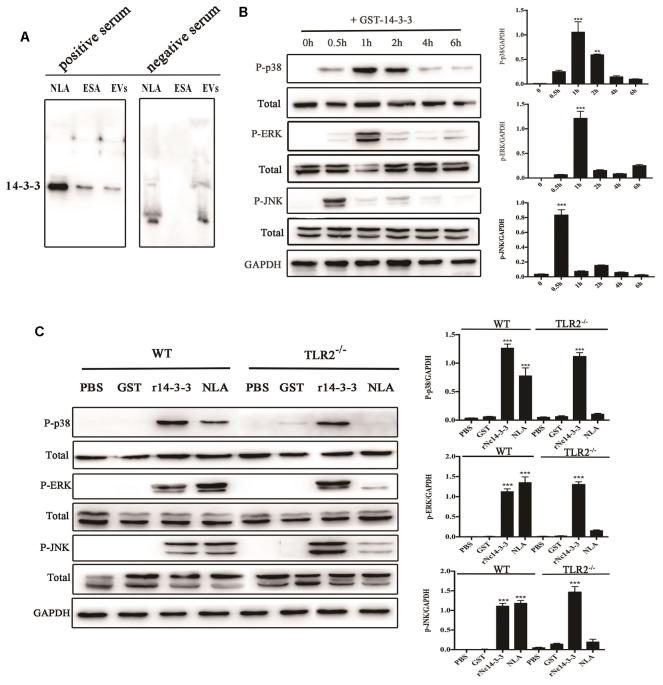
Recombinant Nc14-3-3 activates MAPK signaling pathways in WT and TLR2^-/-^ mouse PMs. **(A)** The 14-3-3 protein detected in different *N. caninum*-derived polypeptides fractions via Western blotting. **(B)** The phosphorylation levels of p38, ERK, and JNK were determined by Western blotting, the phosphorylation of p38 (at 1 h), ERK (at 1 h), and JNK (at 0.5 h) was clearly observed and the relative gray intensity analysis of the Western blotting. **(C)** WT and TLR2^-/-^ mouse PMs were incubated with rNc14-3-3 protein or *N. caninum* lysate antigen (NLA) for 1 or 0.5 h, and the phosphorylation levels of p38 (1 h), ERK (1 h), and JNK (0.5 h) were determined by immunoblot analysis, and the relative levels of the signals from the western blot in panel. ^∗^*P* < 0.05; ^∗∗^*P* < 0.01; ^∗∗∗^*P* < 0.001 for the rNc14-3-3 group versus the PBS group.

LAL assays showed that the concentration of endotoxin in rNc14-3-3 was 0.03 EU/ml, which could not induce TLR2/TLR4 activation or the production of IL-8 or TNF-α (Nozoe et al., 2017; Teodorowicz et al., 2017). Our previous work demonstrated that mouse bone marrow-derived macrophages (BMDMs) exposed to *N. caninum* tachyzoites, NLA, ESA, and EVs can induce the phosphorylation of MAP kinases 30 min after treatment (Li et al., 2018). These data suggested that *N. caninum* activates predominantly the MAPK signaling pathway, probably through a component of its ESA or EV proteins. We hypothesized that rNc14-3-3 participates in the activation of the MAPK signaling pathway. First, Western blotting analysis using the anti-14-3-3 antibody detected the 14-3-3 protein in *N. caninum*-derived polypeptides, including NLA, ESA, and EVs (Figure 4A). We hypothesized that rNc14-3-3 participates in the activation of the MAPK signaling pathway. Specifically, we examined the phosphorylation of MAP kinases in mouse PMs after treatment with rNc14-3-3. WT mouse PMs were treated with 50 μg/ml rNc14-3-3 for the indicated times, and the results revealed that rNc14-3-3 induced strong phosphorylation of p38, ERK, and JNK at 1, 1, 0.5 h, respectively; the phosphorylation of these proteins subsequently weakened over time (Figure 4B). To estimate whether rNc14-3-3 induced the phosphorylation of the p38, ERK, and JNK MAP kinases through TLR2, TLR2^-/-^ and WT mouse PMs were treated with rNc14-3-3 for 1 or 0.5 h. The phosphorylation of p38, ERK, and JNK was observed in both WT and TLR2^-/-^ mouse PMs (Figure 4C). These data suggested that rNc14-3-3 induced phosphorylation of p38, ERK, and JNK MAP kinases not by TLR2. In addition, we treatment mouse PMs with *N. caninum* lysate antigen (NLA) as positive control, the results showed that NLA could induce the phosphorylation of p38, ERK, and JNK after treatment for 0.5 h, while the phosphorylation levels of p38, ERK and JNK were significantly reduced in the TLR2^-/-^ cells, which demonstrated that NLA induced phosphorylation of P38, ERK and JNK via TLR2. Considering that the rNc14-3-3 protein was a GST fusion protein, we further proved that the GST-labeled protein had no effect on our results (Figure 4C).

**FIGURE 5:**
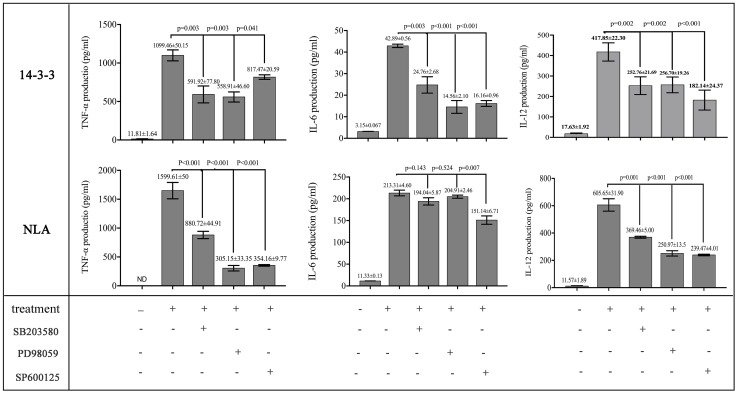
Recombinant Nc14-3-3-mediated cytokine production was decreased after blocking the MAPK signaling pathways. WT mouse PMs were pretreated with or without the SB203580 (p38), PD98059 (ERK) and SP600125 (JNK) inhibitors before incubated with rNc14-3-3 or *N. caninum* lysate antigen (NLA) for 24 h. The production of IL-6, IL-12p40, and TNF-α in the supernatants was measured by ELISA. Data are expressed as the mean ± SD from three separate experiments. ^∗^*P* < 0.05; ^∗∗^*P* < 0.01; ^∗∗∗^*P* < 0.001 for the inhibitor-treated group versus the Nc14-3-3 group or the inhibitor-treated group versus the NLA group.

To obtain more information about the effects of rNc14-3-3 on the *N. caninum-*induced phosphorylation of MAP kinases, we used the MAPK inhibitors SB203580 (p38), PD98059 (ERK), and SP600125 (JNK). WT mouse PMs were pretreated with or without inhibitors for the indicated time at 37°C and then incubated with 50 μg/ml rNc14-3-3 or NLA for 24 h. ELISA demonstrated that PMs treated with the inhibitors could significantly decrease the production of IL-12p40 and TNF-α, whether rNc14-3-3 or NLA. In contrast, significantly less production of IL-6 was observed in rNc14-3-3 cells treated with p38, ERK and JNK inhibitors, while no significant difference in NLA cells treated with p38 and ERK inhibitor (Figure 5). We also detected the production of IL-1β, but the expression of this factor was almost undetectable (data not shown).

### Recombinant Nc14-3-3 Induced Cytokine Secretion by Activating the AKT Pathway

**FIGURE 6:**
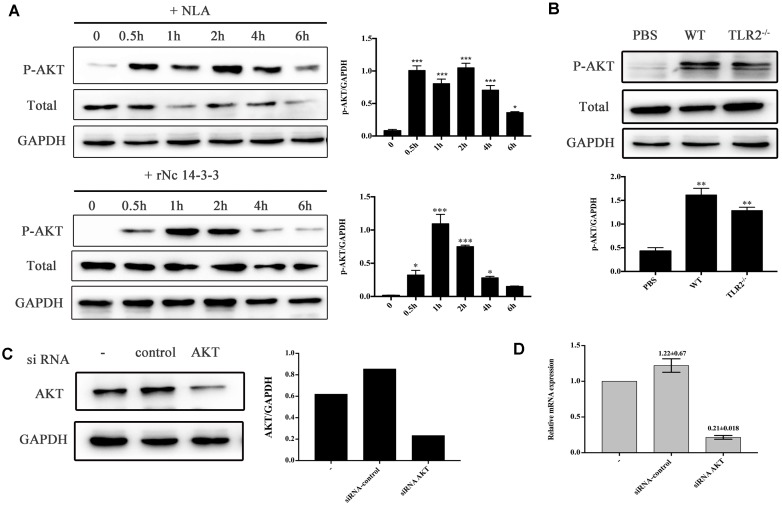
Recombinant Nc14-3-3 activates AKT signaling pathways in WT and TLR2^-/-^ mouse PMs. **(A)** The phosphorylation levels of AKT in WT mouse PMs were determined by Western blotting after rNc14-3-3 or NLA treated for the indicated times. The phosphorylation of AKT was significantly increased at 1 h and the relative levels of the signals from the western blotting in panel. **(B)** The production of phosphorylation AKT in the WT and TLR2^-/-^ mouse PMs was measured by Western blotting after incubated with rNc14-3-3 protein for 1 h and the relative gray intensity analysis of the Western blotting. **(C)** WT mouse PMs were transfected with a small-interfering RNA targeting AKT for 24 h, and AKT protein levels were significantly reduced after RNA interference, as detected by Western blotting and the relative gray intensity analysis of the Western blotting. **(D)** The observed changes in AKT RNA levels after siRNA interference were consistent with protein levels.^∗^Data are expressed as the mean ± SD from three separate experiments. ^∗^*P* < 0.05; ^∗∗^*P* < 0.01; ^∗∗∗^*P* < 0.001 for the treated groups versus the untreated or PBS group.

To investigate whether rNc14-3-3 was important for inducing the activation of AKT in *N. caninum* infection, we employed Western blotting and siRNA interference technology. The results showed that rNc14-3-3 induced a remarkable phosphorylation of AKT after stimulation for 1 h, and there was minimal phosphorylation in negative control cells (Figure 6A, down). *N. caninum* lysate antigen (NLA) could induced the phosphorylation of AKT, the phosphorylated AKT peaked at 2 h and then returned to weaken (Figure 6A, up). To estimate whether rNc14-3-3 induced the phosphorylation of AKT through TLR2, WT and TLR2^-/-^ mouse PMs were treated with rNc14-3-3 or PBS as a negative control. AKT phosphorylation was detected in both WT and TLR2^-/-^ mouse PMs (Figure 6B), suggesting that rNc14-3-3 induced the activation of AKT but had little relationship with TLR2.

**FIGURE 7:**
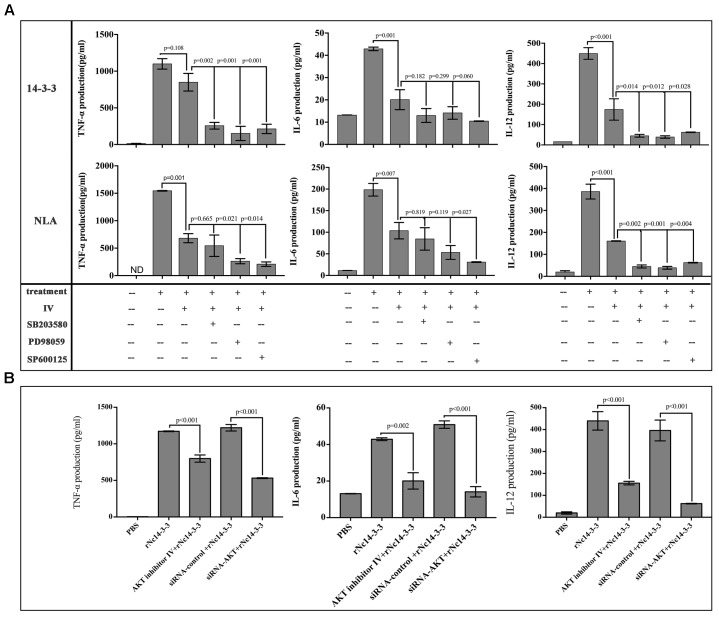
The production of inflammatory cytokines was significantly reduced after blocking the AKT signaling pathway. **(A)** WT mouse PMs were pretreated with the MAP kinases inhibitors in addition or not AKT inhibitor IV before rNc14-3-3 or NLA treatment for 24 h, the production of IL-6, IL-12p40, and TNF-α in the supernatants was measured by ELISA. **(B)** WT mouse PMs were pretreated with AKT inhibitor IV or transfected with a small-interfering RNA targeting AKT for 24 h and then further stimulated with rNc14-3-3 for 24 h. The supernatants were removed and assayed for IL-6, IL-12p70, and TNF-α using ELISA. Data are expressed as the mean ± SD from three separate experiments. ^∗^*P* < 0.05; ^∗∗^*P* < 0.01; ^∗∗∗^*P* < 0.001 for the inhibitor-treated group versus the Nc14-3-3 group or the inhibitor-treated group versus the NLA group.

To confirm this result, WT PMs were transfected with siRNA-control or siRNA-AKT for 24 h using the Lipofectamine 2000 transfection reagent. Following transfection, the cells were further stimulated with 50 μg/ml rNc14-3-3 for 24 h. AKT expression levels were detected by Western blotting and reverse transcription (RT)-PCR, and the results showed that siRNA-AKT significantly reduced AKT expression (Figures 6C,D). Next, we attempted to identify the role of the AKT signaling pathway in the regulation of inflammatory cytokines when mouse PMs were exposed to rNc14-3-3. The results showed that AKT inhibitor IV significantly blocked the production of IL-6, IL-12p40 and TNF-α, similar results were found in NLA treatment group (Figure 7A). In addition, siRNA-AKT also significantly reduced the production of IL-6, IL-12p70 and TNF-α, which was consistent with the results obtained with the AKT inhibitor (Figure 7B).

### Recombinant Nc14-3-3 Induced the Translocation of the NF-κB/p65 Subunit to the Nucleus

**FIGURE 8:**
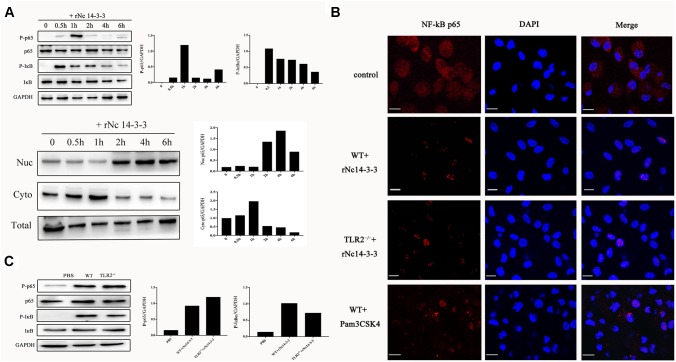
Recombinant Nc14-3-3 induces the translocation of NF-κB/p65 subunits to the nucleus. (**A**, Top) WT mouse PMs were incubated with rNc14-3-3 for the indicated times and the phosphorylation levels of p65 and IκB were determined by Western blotting and relative gray intensity analysis. (Bottom) WT mouse PMs were incubated with rNc14-3-3; then, cytoplasmic and nuclear proteins were extracted separately. NF-κB/p65 protein levels were reduced in the cytoplasm but increased correspondingly in the nucleus at 2 h. **(B)** The effects of rNc14-3-3 or Pam3CSK4 on the translocation of NF-κB/p65 from the cytoplasm to the nucleus in the WT and TLR2^-/-^ group were observed after incubation with rNc14-3-3 through laser scanning confocal microscopy. **(C)** WT and TLR2^-/-^ mouse PMs were incubated with 50 μg/ml of the rNc14-3-3 protein for 1 h, and the phosphorylation levels of p65 and IκB were determined by Western blotting and relative gray intensity analysis.

When NF-κB/p65 is activated, it translocates from the cytoplasm to the nucleus (Inta et al., 2006), and its transcriptional activity is also regulated by phosphorylation by serine/threonine kinases (Ryu et al., 2011). Thus, we isolated the total protein, the cytoplasmic fraction and the nuclear fraction. As shown in Figure 8A, phosphorylated NF-κB/p65 was observed in WT PMs treated with rNc14-3-3, and the protein level of NF-κB/p65 was reduced in the cytoplasm but increased correspondingly in the nucleus at 2 h. As the degradation of IκBα is required for NF-κB activation, we also examined the changes in phosphorylated IκBα. The level of IκBa in WT PMs was significantly reduced after exposed to rNc14-3-3 for the indicated times. To further clarify whether NF-κB/p65 translocates from the cytoplasm to the nucleus, we used laser scanning confocal microscopy, as shown in Figure 8B, NF-κB/p65 was located in the cytoplasm in the control group and distributed mainly in the nucleus after rNc14-3-3 treatment for 2 h. In addition, the expression levels of NF-κB/p65 and IκBα in TLR2^-/-^ mouse PMs were similar to those in WT mouse PMs (Figure 8C). Taken together, our results indicated that rNc14-3-3 could induce the translocation of NF-κB from the cytosol to the nucleus during *N. caninum* infection.

## Discussion

*Neospora caninum* is an intracellular parasite that causes significant economic losses in the cattle industry; although many control measures have been suggested, these strategies have been ineffective (Dubey et al., 2007; Reichel et al., 2015), and the immune mechanisms of the parasite-host interaction are not yet fully understood (Jin et al., 2017b). In our study, we established that *N. caninum* 14-3-3 protein can induce effective immune responses and stimulate cytokine expression by activating the MAPK, AKT, and NF-κB signaling pathways, suggesting that rNc14-3-3 is a novel vaccine candidate against neosporosis.

Macrophages, along with dendritic cells (DCs), play a key role in pathogen elimination and control the steady state of the internal environment as the first line of cell-mediated defense (Hecker et al., 2015). Macrophage functions include the synthesis of cytokines and costimulatory molecules and the presentation of antigens to T cells through the major histocompatibility complex (MHC), which triggers the adaptive immune response (Monney and Hemphill, 2014). The production of inflammatory cytokines is initiated by the recognition of highly conserved sets of molecular patterns (PAMPs) by PRRs (Jenkins et al., 2010). Previous studies have shown that the TLR2, TLR3, and TLR11 innate recognition pathways trigger inflammatory responses to control *N. caninum* infection (Kawai and Akira, 2010; Mineo et al., 2010). However, the interaction of these PAMPs with the PRRs on innate cells that are triggered by *N. caninum* has not yet been fully elucidated. Previous studies have demonstrated that the MAPK pathway acts as a modulatory mechanism during IL-12 production in macrophages after infection with *N. caninum* and *T. gondii*, and this response is dependent on the phosphorylation of p38 MAPK (Quan et al., 2015; Mota et al., 2016). In addition, previous research from our laboratory has shown that *N. caninum* infection causes the upregulation of IL-12p40 in a TLR11- and MEK-ERK activation-dependent manner (Jin et al., 2017a). Therefore, the MAPK pathway plays a key role in the control of intracellular parasite infection, resulting in the regulation of inflammatory cytokines that are responsible for Th1 immune responses, including IFN-α/γ and TNF-α. Our results indicate that *N. caninum* live tachyzoites, along with *N. caninum*-derived proteins (NLA, ESA, and EVs), are capable of inducing MAPK activation. We speculate that *N. caninum* activates the MAPK pathway after the infection of mouse PMs, probably through a component of its ESA, especially proteins included in EVs. Apicomplexan parasites can invade host cells by using the action of secretory organelles, such as micronemes, rhoptries, and dense granules. *Toxoplasma* secretes GRA24, which traffics from the vacuole to the host cell nucleus, then triggers prolonged autophosphorylation and nuclear translocation of the host cell p38α MAP kinase (Braun et al., 2013). However, there are no apparent GRA24 orthologs within the *N. caninum* genome.

The 14-3-3 protein is a widely expressed acidic protein that spontaneously forms dimers and was the first to be identified as a phosphoserine-threonine-binding module (Tzivion and Avruch, 2002). 14-3-3 has been isolated and sequenced in many apicomplexan parasites, including *T. gondii* (Koyama et al., 2001), *N. caninum* (Lally et al., 1996), *E. tenella* (Zhao et al., 2014), and Cryptosporidiidae (Brokx et al., 2011). *T. gondii* 14-3-3 (Tg14-3-3) was detected in tachyzoites and present mainly in the cytoplasm, with a less membrane-associated distribution (Koyama et al., 2001). Recent studies have shown that Tg14-3-3 is present within the parasitophorous vacuolar space and can rapidly recruit host cell 14-3-3 to the PV membrane, inducing the migratory activation of immune cells (Weidner et al., 2016). The gene that encodes 14-3-3 in *N. caninum* exhibits 98.7% identity with the *T. gondii* 14-3-3 sequence, suggesting that both proteins probably represent the same isoform and perform similar functions. Detection by immunofluorescence microscopy showed that Nc14-3-3 was mainly found in the cytosol and cell membrane, consistent with the results reported for *T. gondii*. The 14-3-3 proteins as a conserved regulatory molecules, which expressed in all eukaryotic cells and able to bind a multitude of functionally diverse signaling proteins, including receptors, cytoskeletal proteins, kinases, enzymes, and transcription factors (Fu et al., 2000). Most investigators have speculated that 14-3-3 proteins may play a role in the transmission of regulatory signals related to cell physiology, and these proteins are known to regulate PKC activity and translocation (Aitken et al., 1992; Siles-Lucas Mdel and Gottstein, 2003). Parasite GPI can induce cellular activation through TLR2, initiating the MAPK and NF-κB signaling pathways (Gowda, 2007). Here, we hypothesized that Nc14-3-3 can modulate the host cell immune response process through the MAPK pathway, and we successfully expressed and purified a recombinant fusion protein of Nc14-3-3 (rNc14-3-3). The results revealed that rNc14-3-3 could induce the strong phosphorylation of p38, ERK and JNK in mouse PMs, and this phosphorylation did not require the help of the TLR2 receptor. The secretion of inflammatory cytokines is helpful for eliminating the parasites, and TNF-α and IL-12 acted as the key mediators of resistance to *N. caninum* by promoting intracellular mechanisms to kill the parasites and inhibit their replication. rNc14-3-3 induced the production of TNF-α, IL-6, and IL-12p70, and this effect was significantly enhanced by p38, ERK and JNK inhibitors, revealing that Nc14-3-3 can serve as a novel effective vaccine for controlling *N. caninum* infection.

The AKT signaling pathway is activated by many combinations of ligands, such as lipopolysaccharide (LPS), TLRs and various cytokine receptors (Manukyan et al., 2010). The activation of the AKT kinase pathway is conducive to cell survival, proliferation, protein synthesis, and other activities that are important for cellular survival and homeostasis (Manning and Cantley, 2007). A parasite-derived neurotrophic factor (PDNF) of *Trypanosoma cruzi* can target host cell AKT as an intracellular antiapoptotic strategy (Chuenkova and PereiraPerrin, 2009). Our results showed that rNc14-3-3 induced the phosphorylation of AKT in WT and TLR2^-/-^ mouse PMs, and inflammatory cytokine levels were reduced after treatment with AKT inhibitor IV and siRNA-AKT. These results suggest that rNc14-3-3 could regulate the production of cytokines dependent on the AKT signaling pathway. Studies have shown that activated AKT promotes cell survival through crosstalk with NF-κB (Romashkova and Makarov, 1999). The invasion of many pathogens causes activation of the transcription factor NF-κB, which plays an important role in initiating the innate immune response (Hayden and Ghosh, 2012). The activation of NF-κB/p65 has been widely reported in several infections and can be mediated by TLR2 (Chen et al., 2012); this activation is mediated by the degradation of IκBα. Previous research indicated that *L. amazonensis* infection can activate p50/p50 NF-κB and depends on PI3K/AKT activation (Calegari-Silva et al., 2015). Here, we demonstrated that in both TLR2^-/-^ and WT mouse PMs, the level of IκBα was reduced, and NF-κB/p65 accumulated in the nucleus after treatment with rNc14-3-3, suggested that rNc14-3-3 activated the NF-κB signaling pathway, which in turn led to increased cytokine production in both TLR2^-/-^ and WT mouse macrophages, implied that TLR2 was not involved in the regulation of rNc14-3-3-induced translocation of NF-κB from the cytosol to the nucleus.

In summary, we demonstrated here that the 14-3-3 protein of *N. caninum* could induce the activation of the AKT, MAPK, and NF-κB/p65 signaling pathways to modulate the immune responses of mouse PMs, suggested that Nc14-3-3 is a good vaccine candidate target for the prevention of neosporosis.

## Ethics Statement

All animal experimental procedures were performed in strict accordance with the Regulations for the Administration of Affairs Concerning Experimental Animals approved through the State Council of China (1988.11.1) and with the approval of the Animal Welfare and Research Ethics Committee at Jilin University (IACUC Permit No. 201711035).

## Author Contributions

SL, PG, XCZ, and JL drafted the main manuscript and performed the data analysis. SL, LT, and XW planned and performed the experiments. SL, XL, PG, and JL were responsible for the experimental design. SL, NZ, JY, XQZ, JL, and XCZ were responsible for guiding and supporting the experiments and revising the manuscript. All authors read and approved the final manuscript.

## Conflict of Interest Statement

The authors declare that the research was conducted in the absence of any commercial or financial relationships that could be construed as a potential conflict of interest.
